# A 3D-printed phantom to validate subject orientation in 3D imaging and recordings

**DOI:** 10.3389/fninf.2026.1613151

**Published:** 2026-05-28

**Authors:** Guillaume Jean-Paul Claude Becq, Olivier Montigon, Sarvenaz Keshmiri, Aurélien Delphin, Paul Galloux, Hervé Mathieu, Benjamin Lemasson, Emmanuel L. Barbier

**Affiliations:** 1 Univ. Grenoble Alpes, CNRS, Grenoble INP, Gipsa-lab, UMR 5216, Grenoble, France; 2 Univ. Grenoble Alpes, Inserm, CHU Grenoble Alpes, CNRS, IRMaGe, Grenoble, France; 3 Univ. Grenoble Alpes, Inserm, UA07 Synchrotron Radiation for Biomedicine, Grenoble, France; 4 Univ. Grenoble Alpes, Inserm, U1216, Grenoble Institut Neurosciences, Grenoble, France

**Keywords:** CT scan, erroneous side, errors in laterality, incorrect side, left-right error, MRI, phantom, right–left error

## Abstract

A phantom defined by a 3D-printed system of markers representing an anatomical coordinate system is proposed. This phantom is designed for use in magnetic resonance and X-ray imaging devices to validate acquisition parameters and prevent laterality errors during image acquisition and processing. The phantom allows visualization of the coordinate system axes – left-right, posterior-anterior, and inferior-superior – in all orthogonal slices of a volume. A computational method, using the phantom as a reference, is also proposed to automatically detect and correct flips and permutations in RAS coordinate system representations. Testing the phantom across four modalities available in our platforms, followed by conversions of the recordings to the NIfTI format, enables the detection, correction, and adjustment of protocols in 4 out of 5 configurations. The 3D printing models and orientation detection/correction code are shared with the community as open-source and open-access resources to enable quick, cost-effective, and accessible production.

## 1 Introduction

**Figure 1:**
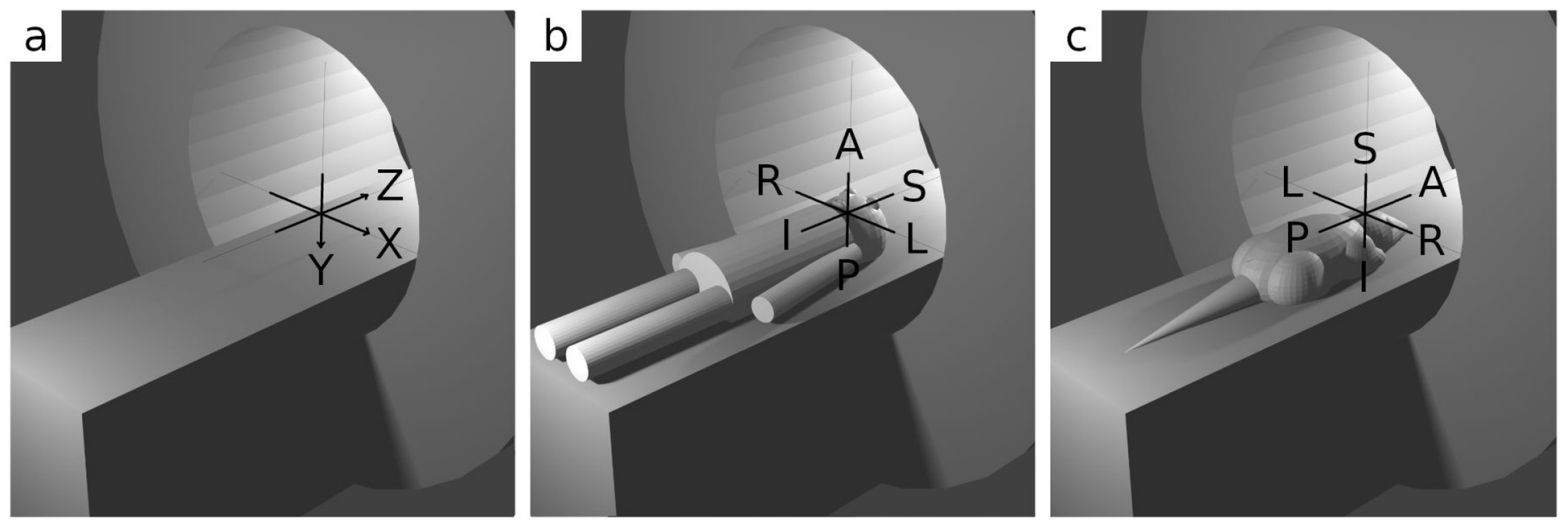
Differences in coordinate systems for: **(a)** the manufacturer's MRI scanner; **(b)** an acquisition with a human subject, a biped in supine position entering head-first into the MRI scanner, i.e., lying on the back with the head toward the back of the device; **(c)** an acquisition with a rodent subject, a quadruped in prone position entering head-first into the MRI scanner, i.e., lying on the belly with the head toward the back of the device.

Ensuring the correct orientation of images obtained after the acquisition of 3D volumes in magnetic resonance imaging (MRI) scanners or computed tomography (CT) scanners can be mind-boggling, with several coordinate systems used by manufacturers, clinicians, radiologists, neurologists, and biologists. The complexity also depends on how the subject is positioned in the device, for practical manipulations or efficient acquisition sequences, as well as the variety of animals with different anatomies, and the diversity of processing software. While it is relatively easy to observe reconstruction or analysis errors on the posterior-anterior axis or the inferior-superior axis, the “left-right flipping is an unfortunately too common occurrence in MRI analysis” (Glen et al., 2020). Although algorithmic solutions are proposed in Glen et al. (2020) and commercial markers may be placed on the body, there appears to be no easy, reliable, inexpensive, and universal system that can validate the orientation across all views during acquisition or processing. These orientation problems become even more subtle when working with different species and subject positions in the device. For example, when working with small animals, subjects are often placed in a prone position, with the head naturally adopting a different orientation than for humans. In Figure 1, three coordinate systems are represented to highlight the differences in coordinate systems that may lead to errors if acquisition parameters are not well configured. In this study, orientations are given according to the convention adopted by NIfTI to share brain images in the right-anterior-superior (RAS) coordinate system, with axis pointing from left (L) to right (R), posterior (P) to anterior (A), and inferior (I) to superior (S) (Cox et al., 2004).

In Figure 1a, there is an example of an X, Y, and Z coordinate system (XYZ) representing the equipment coordinate system of an imaging device. This coordinate system may vary between manufacturers or acquisition modalities. For this proposed setup, assuming the user is in front of the device for positioning the subject into the device, the X-axis is pointing toward the right of the device, the Y-axis toward the bottom, and the Z-axis toward the back. In this coordinate system, if the acquisition is made on a human, a biped animal entering the device head first in the supine position, as represented in Figure 1b, the XYZ system aligns with the anatomical coordinate system of the human in the LPS system, a standard used for DICOM format (Atkinson, 2022; NEMA, National Electrical Manufacturers Association, 1993). If the acquisition is performed on a rodent, a quadruped animal entering the device head first in the prone position, as represented in Figure 1c, the XYZ system corresponds to the RIA system, and transformations are necessary to convert the image into the conventional RAS system adopted by NIfTI.

It should be noted that the recorded data also uses another coordinate system, ijk, representing the indices of the structured array containing the raw data. The link between local parameters and orientations is provided by manufacturers in the metadata. Additional dimensions can also be included, for example, in functional MRI (fMRI) or in arterial spin labeling (ASL) sequences, to represent time. The elements of the array containing the data are generally called voxels, and the relationship between the different maps (ijk) to the device coordinate system or the subject's anatomical coordinate system is defined using affine transforms. With all these orientations and parameters in mind, it is necessary to validate the position of the subject inside the device when developing software libraries or when processing data, in order to transform the recorded data into exchangeable files that adhere to the naming conventions, structures or ontologies adopted by communities, such as DICOM, NIfTI or BIDS (Gorgolewski et al., 2016) in neuroscience. While it is easy to check the antero-posterior or infero-superior axes based on anatomical markers, it can be challenging to maintain a strict control of the orientation throughout the acquisition and processing pipeline for the left-right regions, which may look similar. This is even more difficult when acquisitions have been performed with irregular settings, as is sometimes the case with the DICOM format, where a large number of parameters can be specified (Mustra et al., 2008).

In this work, we propose a small object, a 3D-printed phantom, to assess the orientation of a subject in the MRI scanner and study the influence of both the acquisition parameters and processing tools. Several studies have been conducted using phantoms, for example, to mimic the behavior of flesh, bone, or tumors in the MRI scanner (Filippou and Tsoumpas, 2018), to enable the registration of different modalities, particularly with CT recordings (Wang et al., 1996; Hunsche et al., 2004; Filippou and Tsoumpas, 2018), or to evaluate the displacement and reconstruction errors induced by the MRI scanner (Ramachandran et al., 2021). Manufacturers have their own validation processes, certainly using phantoms, to calibrate devices, ensure the correspondence between acquisition parameters and displayed images, or conform to standards such as exporting images into DICOM format to obtain certifications. Some phantoms have also been developed to establish consensus for quality assurance, for example, to study measurement differences between acquisition centers and devices (Captur et al., 2016). In both cases, calibrations and setups are restrictive and may not be adapted to different devices or processing methods.

In this study, we focus on using an approach that prevents errors in setting acquisition or processing parameters to ensure correct orientations are preserved throughout the entire imaging pipeline. With our approach, the orientations can be validated through simple visual inspection of any acquisition slice, at any step of the processing, as well as with a computational method that detects flips and permutations applied during the acquisition. The transformation required to obtain the valid orientation in the RAS coordinate system can then be quickly computed. The proposed phantom can be obtained from 3D printers readily available in labs, for example, for making specific animal cradles or coils fixation devices with cost and time savings (Herrmann et al., 2014). The article is organized as follows: First, the materials and methods used to construct the phantom and validate the acquisitions on different imaging devices are presented. Second, the results of acquisitions and corrections done after conversions from different software are presented. Finally, the insights obtained and improvements of the proposed phantom are discussed.

## 2 Materials and methods

### 2.1 3D volumetric constructions and printing

The 3D parts of the phantom were designed with Blender (Blender, Online Community, 2018), a well-known 3D computer-graphics software that is free and widely used by various communities. This software allows export in stereolithography format (.STL) that can be imported into the software used by the 3D printing device of the lab. The 3D printer used in this study is an UltiMaker S3 (Ultimaker B.V., Utrecht, Netherlands). It is available to researchers at the Grenoble Institute of Neurosciences for creating custom experimental setups on demand, primarily for positioning or restraining small animals in a device. The phantom was printed with acrylonitrile butadiene styrene (ABS), a low-cost and durable material. The printing parameters were: resolution 0.15 mm (Normal); infill density: 15 %; infill pattern: Grid.

**Figure 2:**
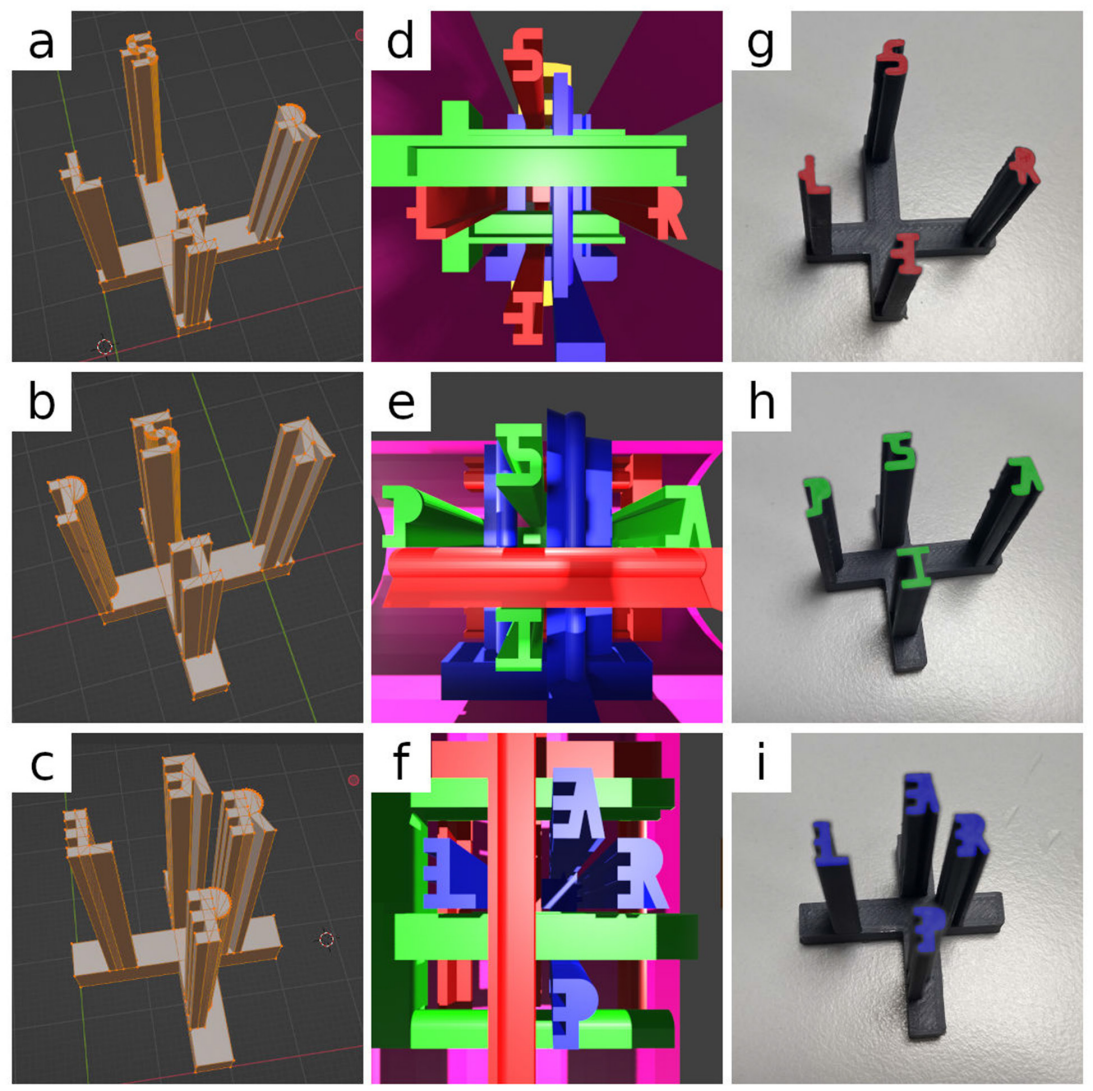
Different views of the three parts of the phantom phantom's components: First row—LR-IS axes for coronal slices; Second row—PA-IS axes for sagittal slices; Third row—LR-PA axes for axial slices. The different modalities are: **(a–c)** modelization with meshes in the mesh-based modeling in 3D software; **(d–f)** renderings in the rendered assemblies of the components in 3D software when the different parts are assembled; **(g–i)** 3D printings. Note the symbols. The annotation keys are: 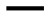, 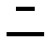, 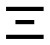, on the left part of the labels correspond to coronal, sagittal and axial planes, respectively. These annotations map to the following markers: 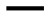 L, 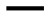 R, 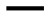 L, 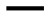
R for left-right axes; 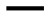
I, 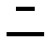
S, 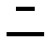 I, 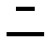 S for inferior-superior axes; 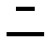
P, 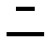 A, 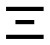 P, 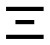
A for anterior-posterior axes.

The proposed phantom consists of three parts that can be printed individually and assembled together. The results of the computer-aided design (CAD) for these three parts are shown in Figures 2a–c.

The structure features a simple design with two crossing bars that form bases supporting four fiducial columns. The columns are created with symbols elevated at different heights. The simplified meshes for each part are also presented in Figures 2a–c. The dimensions of the components, provided in millimeters, are as follows^1^: bases = 30 × 30 × 5; columns = 5 × 5 × 30. When assembled, the internal volume of the system, comprising all three parts, measures 30 × 30 × 30 mm. A symbol indicating the index of the part is added to the letter to discriminate between LR axis in the coronal or axial slices, AP axis in the sagittal or axial slices, IS axis in the coronal or sagittal slices: 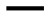 is used for coronal slices, 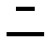 for sagittal slices and 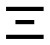 for axial slices^2^. The marker sets are organized as follows: 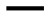 **L** 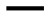 **R** 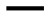 **I** 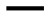 **S** , for markers in the coronal plane; 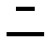 **P** 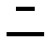 **A** 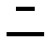 **I** 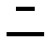 **S** , for markers in the sagittal plane; and 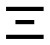 **L** 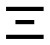 **R** 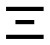 **P** 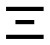 **A** for markers in the axial plane. The virtual assembly of the parts was verified using the 3D computer graphics software. Rotations were applied to align for the different positions on the elevated fiducial marks and alignment of the three parts of the system as illustrated in Figures 2d–f.

**Figure 3:**
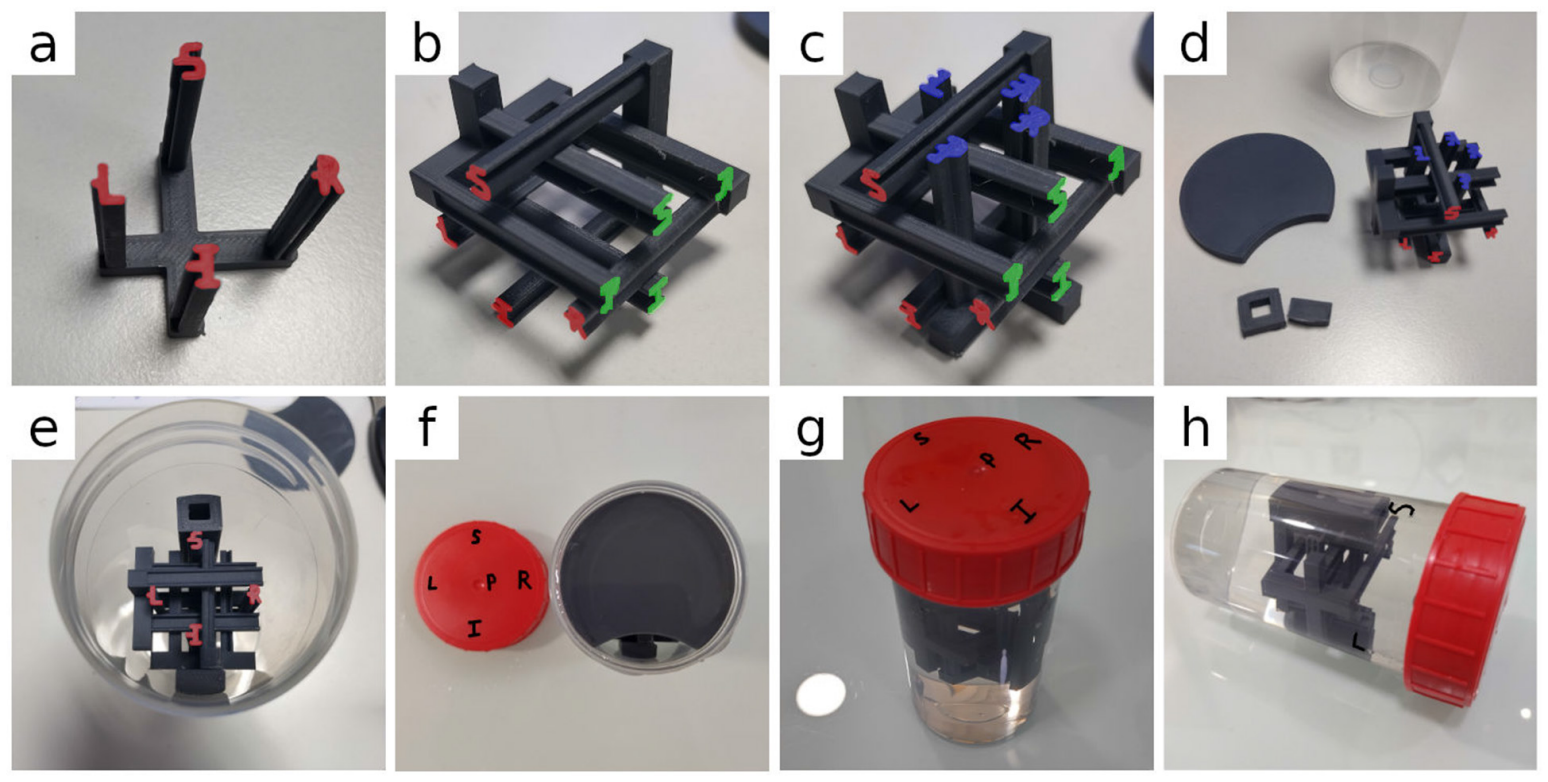
Assembly steps for the phantom: **(a)** Single 3D-printed piece; **(b)** Two assembled pieces; **(c)** Three assembled pieces; **(d)** Full 3d assembly with two blockers, bubble retainer, and cylindrical tube; **(e)** System inserted into the tube; **(f)** The bubble retainer is placed at the top of the cylinder and filled with water for MRI recordings. The hole in the retainer aligns with the superior axis to allow bubble evacuation and trapping between the cap and retainer. **(g, h)** The system is ready for recording, with axis directions labeled on the tube and cap.

The three printed parts are shown in Figures 2g–i and are designed to obtain the anatomical planes or slices commonly used in neurology: coronal slices, which contain the left-right (LR) and inferior-superior (IS) axes; sagittal slices or parasagittal ones, containing the posterior-anterior (PA) and the IS axes; axial or transverse slices, containing the LR and PA axes. The neurological convention is adopted here, i.e., coronal slices are given looking toward the anterior direction, sagittal slices are given looking toward the left direction, and axial slices are given looking toward the inferior direction of the brain. The three parts are printed individually, allowing for layer superposition. This approach avoids the need for complex space-filling techniques or additional printing. The physical assembly of the three parts is illustrated as follows: one part in Figure 3a, two parts in Figure 3b, and three parts in Figure 3c.

The orthogonality and print quality were verified through visual inspection, with no deviation observed from the targeted cubic width of 30 mm (measured with a ruler precise to 1 mm). Additional parts are also printed to allow the system to be positioned in a standard transparent polyethylene cylindrical tube as given in Figures 3d–h. The assembled system within the tube is referred to as the phantom and can be used with various settings for different imaging devices.

### 2.2 Imaging devices and acquisitions

At Grenoble University, several medical devices are shared among the community via the IRMaGE platform (IRMaGe, 0000). Medical devices available for imaging of 3D structures used in this study are: a preclinical 4.7 T MRI scanner (BioSpec 47/40 USR, Bruker) with Paravision 7.0; a microCT scanner for computed tomography (VivaCT40-SCANCO); a portal imager in a Small Animal Radiation Research Platform (SARRP); and a 3.0 T human MRI scanner (MR Systems Achieva dStream Release 5.7 2021-01-08, Philips).

For the 4.7 T MRI, a T2w TurboRARE sequence is used for an acquisition with the following parameters: TR = 9514.71 ms, TE = 22.18 ms, pixelBW = 225.36 Hz, FlipAngle = 180 deg, spatial resolution ≈ 0.391 × 0.391 × 0.391 mm, field of view ≈ 50 × 50 mm, array size = 128 × 128 × 128. Acquisition with the microCT is done using these parameters: voltage 70 kVp, current 112 μA, integration time 200 ms, resolution 76 μm, binning 4, 125 projections / 180°, the maximal field of view being 38.912 mm. Descriptions of the X-ray source and detector for the electronic portal imaging device for 2D radiographic images of the SARRP are given in Anvari et al. (2018), with acquisition parameters remaining the same. For the 3.0 T human MRI Scanner, T1w acquisitions are done with these parameters for coronal acquisitions: TR = 14.7884 ms, TE = 7.323 ms, pixelBW = 89.7569 Hz, FlipAngle = 8 deg, spatial resolution ≈ 0.5 × 1 × 0.5 mm, field of view ≈ 256 × 256 mm, array size = 512 × 256 × 512.

### 2.3 Software used for conversion into the NIfTI format

Volumetric data obtained in raw format from the different recording devices are converted to NIfTI format, either using in-house software developed at GIN like MRI File Manager (Montigon, 2015) and MP3 (Brossard et al., 2020), or other free software developed by other teams, like brkraw (Lee et al., 2020) version 0.3.11, for converting Bruker files, or dcm2niix (Li et al., 2016) version 1.0.20211006, for converting DICOM files into NIfTI ones. Volumes are visualized using MRIcron (Rorden et al., 2007) version 1.2.20211006, or nibabel (Brett et al., 2024) version 5.3.2. If laterality errors are observed or if other orientation issues occur, images are corrected using nibabel for reading data, changing affine matrices, and converting them back to NIfTI files, until the volumes are correctly aligned in the RAS coordinate system.

### 2.4 A method for the automatic detection and correction of flips and orientation errors

Finally, we propose a method for automatically retrieving the flips and permutations that may be artificially introduced and observed during the data acquisition and preprocessing processes. The method relies on simulating all possible configurations of flips and permutations that can be generated from an original template volume representing the phantom in 3D. By matching these simulated configurations with the acquired volume under test, we identify the transformations required for potential corrections. The processing can be decomposed into three steps: (1) constructing templates for phantom analysis using MRI or CT acquisitions; (2) simulating configurations and evaluating matching performance; (3) correcting affine transformations based on the detected configurations.

#### 2.4.1 Generating 3D phantom templates

**Figure 4:**
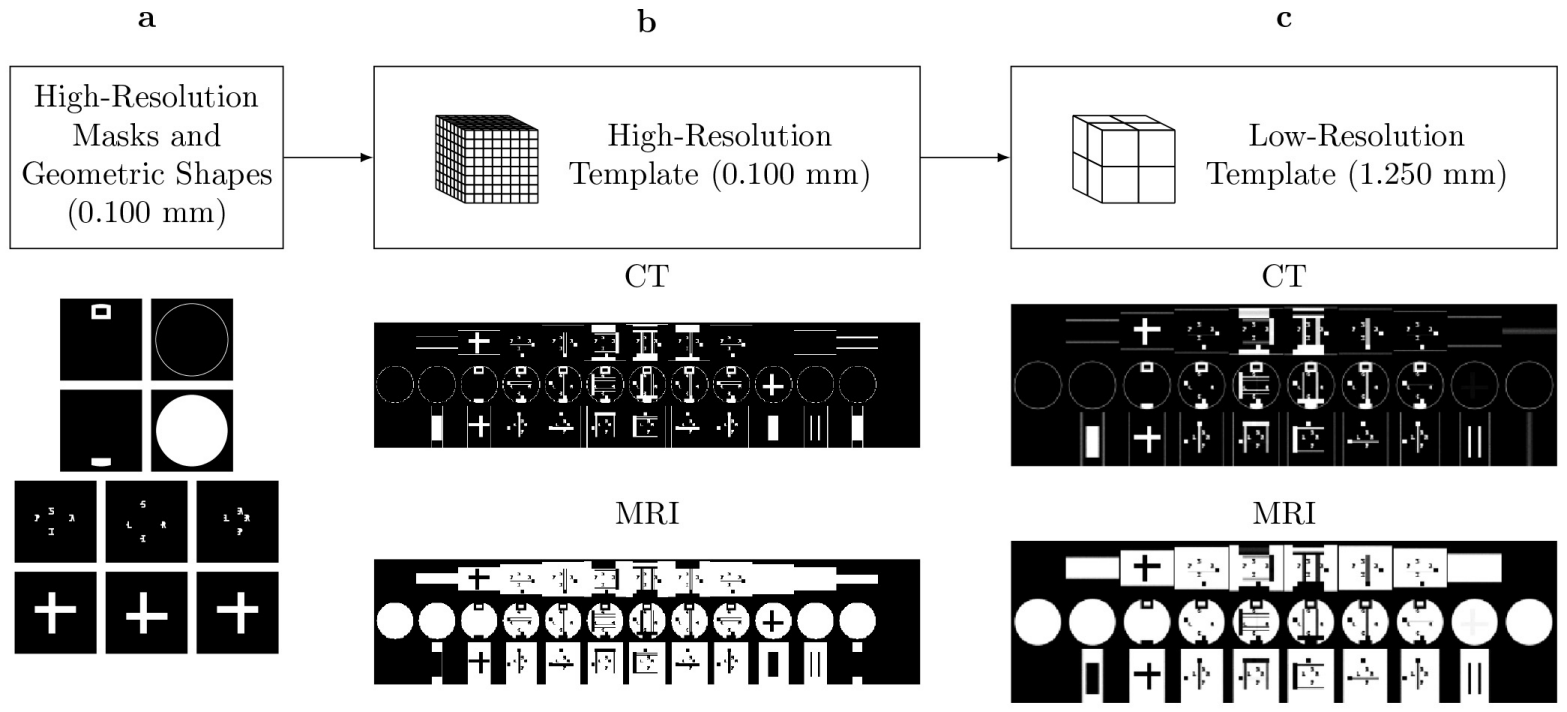
Template creation for automatic flip and orientation detection: **(a)** 3D volumetric phantom components used to generate masks: upper/lower maintainers, tube (empty and water-filled), fiducial markers and bases; **(b)** Masks applied slice-by-slice in all three dimensions to create high-resolution templates for MRI/CT scans, using specific mask combinations; Examples shown for sagittal (top), coronal (middle), and axial (bottom) slices; **(c)** Low-resolution templates generated by random sampling points in a low-resolution voxel and averaging their high-resolution voxel values. Low-resolution templates improve processing speed.

A scheme for creating 3D phantom templates is given in Figure 4.

First, 2D mask images of the different components of the elements printed in 3D are obtained from the sliced objects created in Blender. These masks are combined with different weights to generate MRI or CT reference images for different slices in 3D volumes. The resulting values are stored in NIfTI format in the RAS configuration. The initial resolution of 0.1 mm is used to create templates as given in Figure 4b. However, computations on these volumes, which contain a large number of voxels to test for alignment, are computationally intensive^3^. Therefore, templates at a lower resolution of 1.25 mm are computed as given in Figure 4c to improve speed performance during alignment comparisons^4^.

For subsampling, for each voxel *V*_low, *i*_ in the low resolution volume, 1,000 random points are drawn with coordinates uniformly sampled in [*x*min, *x*max] × [*y*min, *y*max] × [*z*min, *z*max] where *x*_min_, *x*_max_, *y*_min_, *y*_max_, *z*_min_, *z*_max_ are the bounding coordinates of the orthogonal cubic voxel. The intensities of these random samples in the corresponding high-resolution voxels *V*_high, *k*_ are averaged and the result is stored in *V*_low, *i*_.

#### 2.4.2 Automatic detection of flip and permutation errors

**Figure 5:**
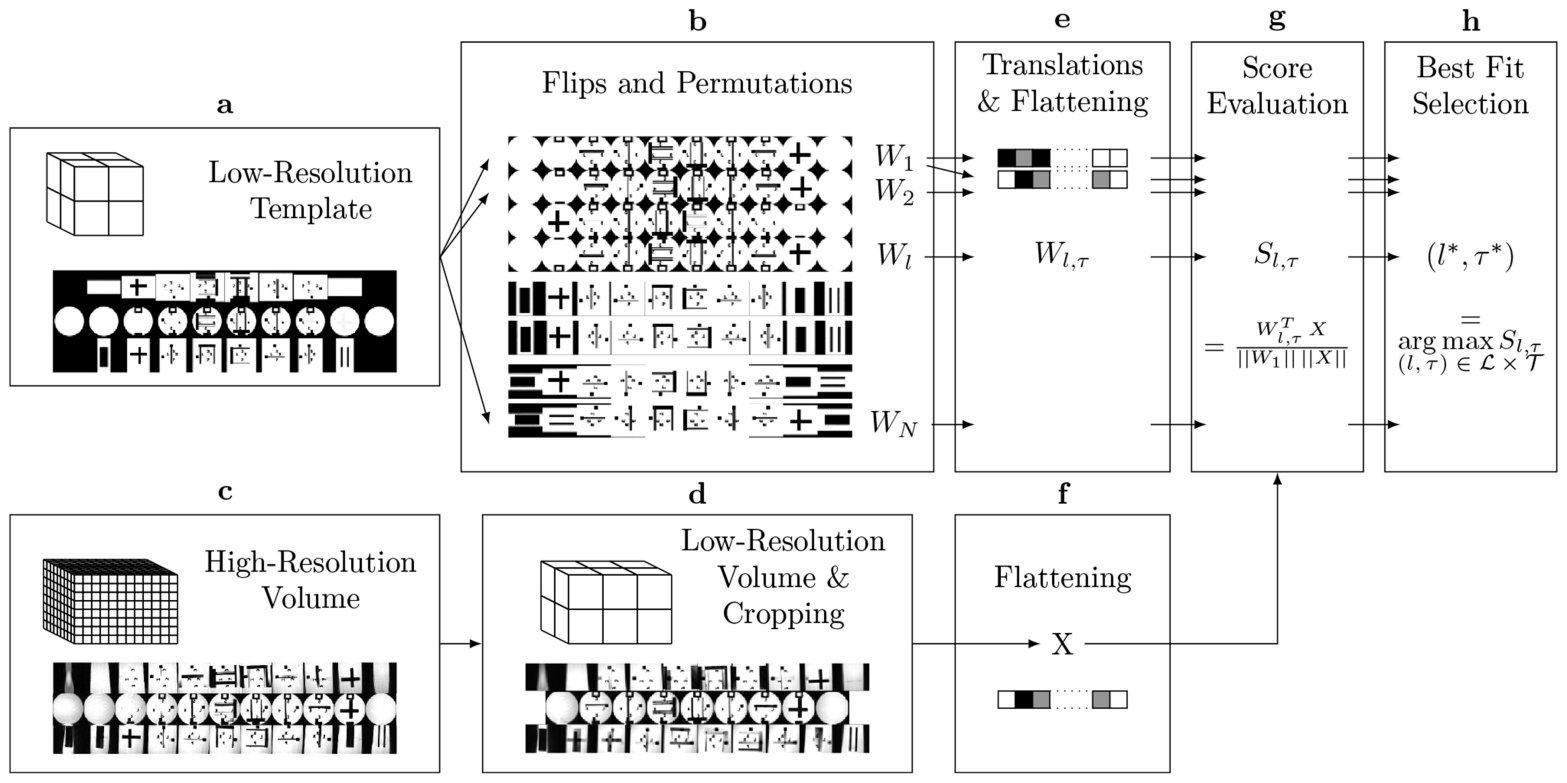
Automatic detection of flips and orientations in MRI acquisitions: **(a)** Reference volume derived from low-resolution MRI template. **(b)** All possible data arrangements generated by flipping and permuting axes from the reference volume, yielding *N* configurations *W*_*l*_; **(c)** The high-resolution volume containing the phantom under detection; **(d)** The analyzed volume is subsampled to match the template's lower resolution and cropped/adjusted to enable translational alignment; **(e)** Template configurations are translated in three directions to generate *T* new configurations *V*_*l*, τ_ per *V*_*l*_, and volumes are flattened into vectors *W*_*l*, τ_. **(f)** The analyzed volume is flattened into vector *X* for comparison with *W*_*l*, τ_. **(g)** A cosine similarity score, computed efficiently via scalar product, is evaluated for each configuration; **(h)** The configuration that obtained the best score indicated the configuration of flips and permutations observed on the analyzed volume.

The low-resolution templates of the 3D phantom are used to generate all possible configurations of artifacts, including those obtained through data flips and axis permutations. These configurations are then matched with the acquired data to identify and classify potential anomalies. A schematic representation of the method is provided in Figure 5.

Considering a flip as the ordering of data in the opposite direction, such as the left-right flipping of data, which is the initial aim of this work, there are 2^3^ = 8 combinations of flips that can be obtained in our configuration. This will be denoted as (*f*_1_, *f*_2_, *f*_3_) where *f*_*i*_ = 1 if there is a flip on the *i*th dimension and *f*_*i*_ = 0 if there is no flip. There are also 3! = 6 possible permutations of the axes. Permutations will be denoted as (*p*(1), *p*(2), *p*(3)), where *j* = *p*(*i*) represents the permutation of axis *i*. For example, a permutation of axes 2 and 3 will be denoted as (1, 3, 2). Flips are performed first, followed by permutations. The independent combinations of flips and permutations lead to *N* = 8 × 6 = 48 possible configurations that can be observed on acquired volumes. These configurations are simulated from the original template, starting from the original configuration *V*_1_ = ((0, 0, 0), (1, 2, 3)) with no flips and permutations. Examples of simulated configurations are given in Figure 5b. In order to account for misalignment with the volume to be tested, additional translations are also simulated to obtain further configurations. Translations are applied in the three dimensions and will be denoted as (τ_1_, τ_2_, τ_3_). The volumes are then flattened and represented as vectors for comparison. This is illustrated in Figures 5e, f to obtain *W*_*l*, τ_ for a configuration and *X* for the volume to be tested. The similarity of the two vectors is evaluated with the cosine similarity, given by $\cos\theta =\frac{W_{l,\tau }^{T}X}{||W_{l,\tau }||||X||}$, where .^*T*^ is the transpose operator. Since all configurations are derived from *W*_1, 1_ with τ = 1 denoting the initial configuration *V*_1_ with no translation, all norms are equal. The optimal value identifies the optimal configuration that best matches the analyzed volume.

#### 2.4.3 Correction of affine transforms using detected flips and permutations

Only the flip and permutation components of the best configuration are retained to correct the original affine transform. Let *A*_1_ be the affine transform associated with the analyzed volume. Let *B*_1_ be the restriction of *A*_1_ to its first three rows and columns, with the values in *A*_1_ replaced by values in the set {−1, 1}. Regardless of the resolution and coordinates of the system, the affine transform associated with the detection performed in the preceding subsection can be denoted as *A*_2_. The flips and permutations matrix *B*_2_ is such that *B*_2*i, j*_ = *f*(*j*) if *j* = *p*(*i*) and *B*_2*i, j*_ = 0 otherwise. Here, *f*(*j*) = −1 if a flip is applied on axis *j* and *f*(*j*) = 1 if there is no flip. In other words, if Π, is a permutation matrix, with Π_*ij*_ = 1 if *j* = *p*(*i*) and 0 otherwise, and *F* a flips matrix, with diagonal elements given by *F*_*ii*_ = *f*(*i*) and zeros elsewhere, then $B_{2}=\text{Π}F=\left(\begin{array}{ccc} {\text{Π}}_{11} & {\text{Π}}_{12} & {\text{Π}}_{13}\\ {\text{Π}}_{21} & {\text{Π}}_{22} & {\text{Π}}_{23}\\ {\text{Π}}_{31} & {\text{Π}}_{32} & {\text{Π}}_{33} \end{array}\right)\left(\begin{array}{ccc} f\left(1\right) & 0 & 0\\ 0 & f\left(2\right) & 0\\ 0 & 0 & f\left(3\right) \end{array}\right)$. Let *P* be the transformation matrix such that *B*_2_ = *PB*_1_, then *P* can be computed as $P=B_{2}B_{1}^{-1}$ where $B_{1}^{-1}$ is the inverse of *B*_1_. This matrix always exists since *B*_1_ is a member of the orthogonal group O(3). The matrix *P* is then extended to an affine transform such that $A_{3}=\left(\begin{array}{cc} P & 0_{3\times 1}\\ 0_{1\times 3} & 1_{1\times 1} \end{array}\right)$. One possible correction for the affine transform is then *A*_1*c*_ = *A*_3_*A*_1_, but this transformation does not account for the center of the volume. A better solution, which preserves the center of the volume, can be obtained by: 1. Removing the translation to the center of the volume, yielding *A*_1*b*_; 2. Applying the flips and permutations to obtain *A*_1*d*_ = *A*_3_*A*_1*b*_; 3. Reapplying the translation to the corrected center, if this coordinate is to be retained.

## 3 Results

### 3.1 Acquisition using the 4.7 T MRI scanner for small animals

To test the phantom, the tube is filled with water and gently tapped to evacuate micro air bubbles. This is achieved by inserting a 3D-printed cover disk with a small section removed, at the top of the tube (Figure 3f) before sealing it. This component allows for capturing any remaining air bubbles in the compartment between the cover disk and the cap, thereby preventing artifacts during acquisition.

**Figure 6:**
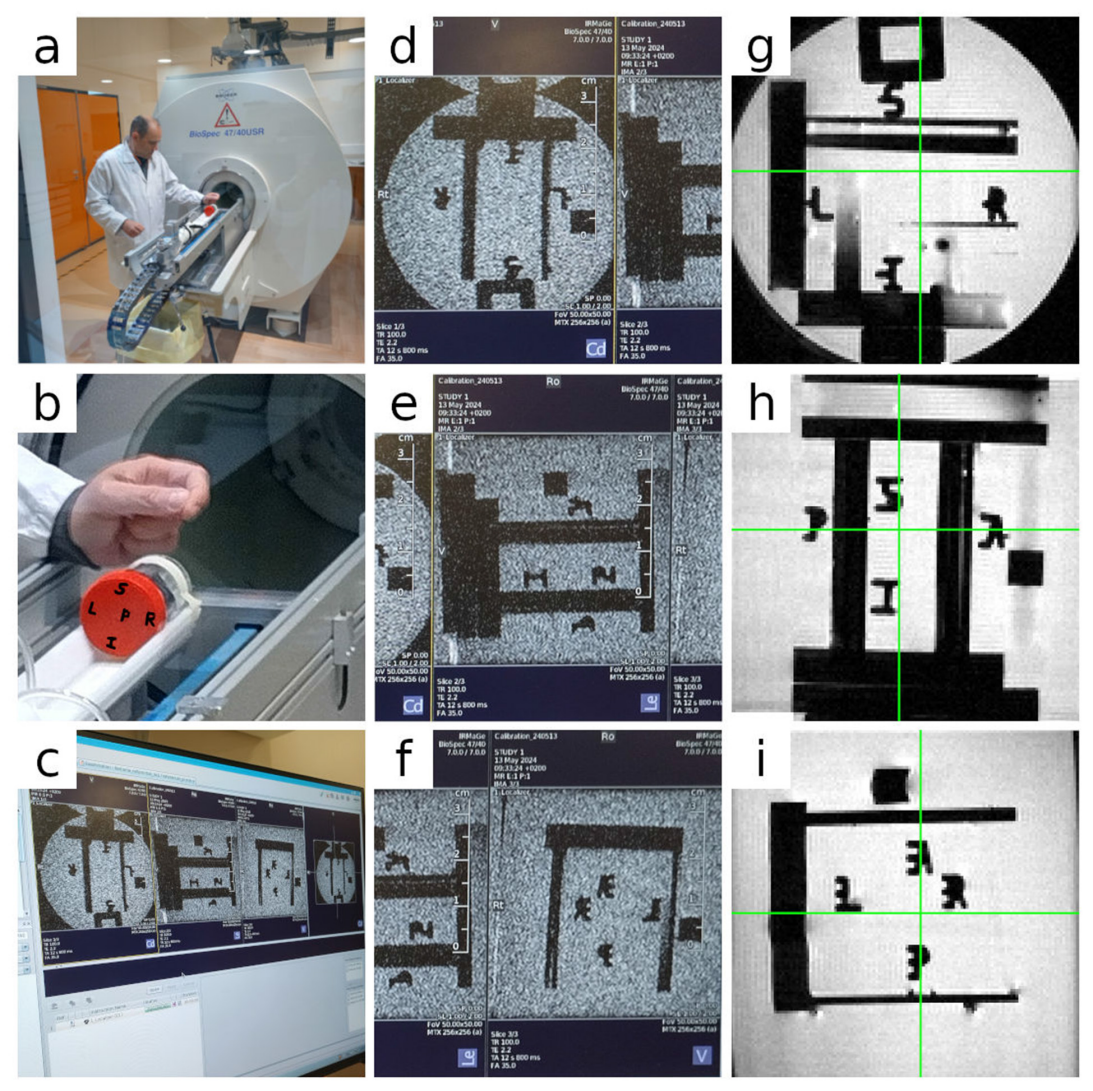
MRI acquisitions using a 4.7 T scanner for small animals: **(a, b)** Phantom positioned in the MRI to mimic a tail-first prone acquisition for quadrupeds; **(c–f)** On-site visualization Paravision acquisition software; **(g–i)** Visualization of the exported NIfTI file, obtained using brkraw, with nibabel.

Fiducial marks indicating the direction of the axes are placed on the curved surface of the tube, and on the top of the cap (Figures 3f–h). The tube is then positioned in the MRI scanner, in the same orientation as a small animal, specifically in the prone, head-first quadruped position (Figures 6a, b).

The first images obtained from the acquisition software, Paravision 7.0, are shown in Figure 6c. Zoomed-in views of the acquisition screen are shown in Figures 6d–f. The images are displayed using an inverted convention^5^ for the axes and their abbreviations: left (Le) - right (Rt) for the LR axis, caudal (Ca) - rostral (Ro) for the PA axis, and ventral (Ve) - dorsal (Do) for the IS axis. While the inverted visualization proposed in Paravision may cause confusion and errors with anatomical images, the proposed phantom allows verification that the acquisition parameters are valid, ensuring that left corresponds to left, ventral corresponds to inferior, and caudal corresponds to posterior, as demonstrated in this study. Parameters in Paravision are also set for acquiring images of a quadruped subject in a head-first, prone position within the device. Raw data from the Bruker device are then exported into a NIfTI file using the Python package brkraw. The resulting file contains the affine transform *A*:


$$\begin{array}{l} A=\left(\begin{array}{cccc} -0.391 & 0 & 0 & 23\\ 0 & 0 & 0.391 & -27.829\\ 0 & 0.391 & 0 & -24\\ 0 & 0 & 0 & 1 \end{array}\right) \end{array}$$

which enables the visualization of the expected coronal, sagittal, and axial slices, for example, using nibabel.niftiImage.orthoview() (Brett et al., 2024) as shown in Figures 6g–i. The acquisition and conversion into the NIfTI format can be validated, and the file can be shared with the community in this exchangeable format without any ambiguity. This acquisition also helped identify and correct a bug in MRI File Manager software (Montigon, 2015) and one in the nibabel.niftiImage.orthoview() function (Brett et al., 2024) to achieve the correct visualization.

### 3.2 Acquisition using the microCT scanner for small animals

**Figure 7:**
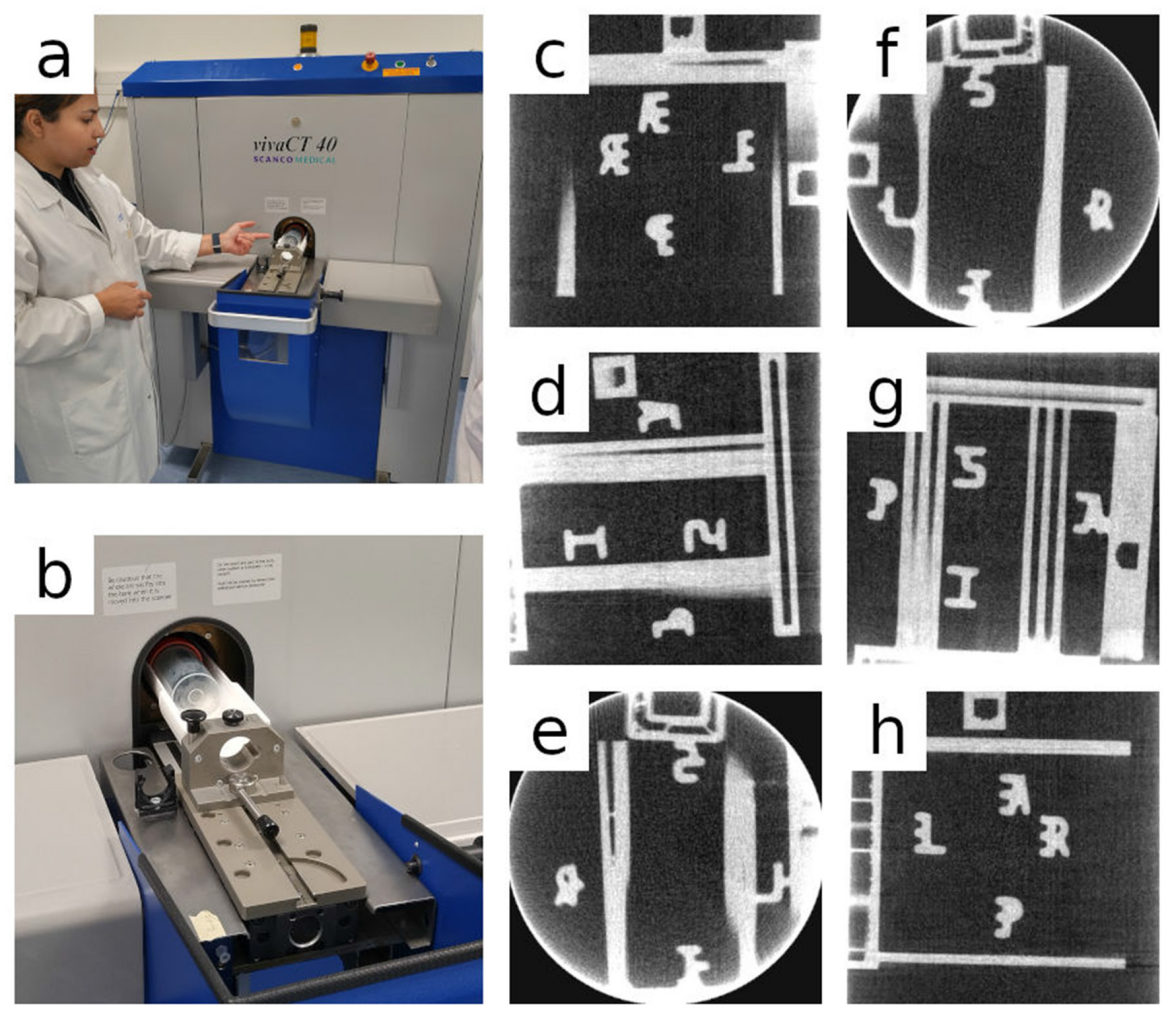
Acquisitions using the microCT scanner for computed tomography: **(a, b)** Phantom positioned to mimic a tail-first prone acquisition for quadrupeds; **(c–e)** Visualization of slices from the initial NIfTI data. **(f–h)** Visualization of slices after orientation correction.

For this modality, the tube containing the phantom is kept dry to avoid artifacts. Following the conventions of the team using the platform, the tube is inserted posterior-first, mimicking an animal inserted in the device with the tail-first, in a prone position, as shown in Figures 7a, b.

Data are recorded as a series of DICOM files and are converted using dcm2niix to obtain an initial version of a NIfTI file. Coronal, sagittal, and axial slices of this recording visualized using MRIcron software are shown in Figures 7c–e, respectively. Let *A*_1_ be the initial affine transform given in the NIfTI file, as given in Equation 1:


$$\begin{array}{r} A_{1}=\left(\begin{array}{cccc} -1 & 0 & 0 & 0\\ 0 & 1 & 0 & 0\\ 0 & 0 & 1 & 0\\ 0 & 0 & 0 & 1 \end{array}\right),\;A_{2}=\left(\begin{array}{cccc} -1 & 0 & 0 & 0\\ 0 & 0 & 1 & 0\\ 0 & 1 & 0 & 0\\ 0 & 0 & 0 & 1 \end{array}\right),\\ A_{3}=\left(\begin{array}{cccc} 1 & 0 & 0 & 0\\ 0 & 0 & 1 & 0\\ 0 & 1 & 0 & 0\\ 0 & 0 & 0 & 1 \end{array}\right) \end{array}$$ (1)

Permutations to correct for the RAS orientation are performed using nibabel and a new file is written with the affine transform *A*_2_, as given in Equation 1. The new slices are in the correct orientation as shown in Figures 7f–h. Finally, a correction was necessary to convert the transformation obtained from the DICOM recordings to the NIfTI format, which is initially set for the acquisition of a biped supine subject and not adapted to the acquisition of a quadruped prone subject.

### 3.3 Acquisition using the electronic portal imaging device of the SARRP

**Figure 8:**
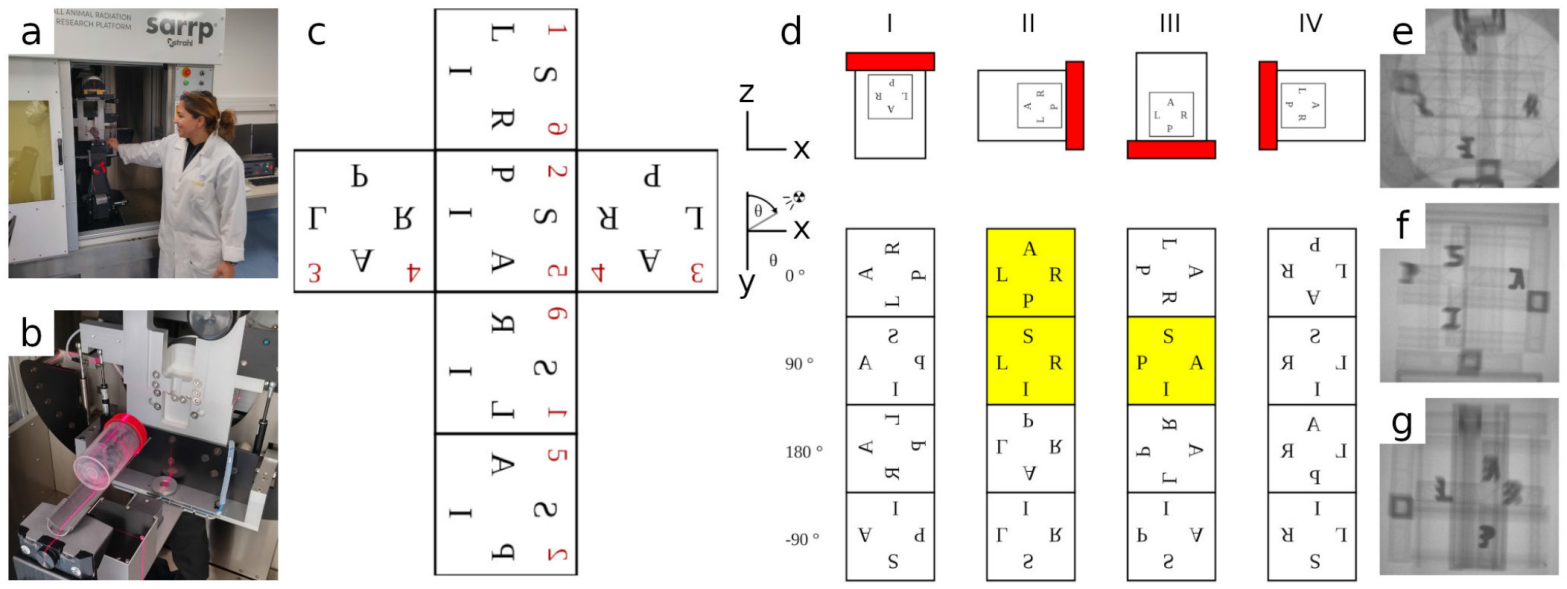
Acquisitions using the SARRP X-ray device: **(a, b)** Phantom mimicking the subject positioned within the device; **(c)** Cube projection of the phantom illustrating the different views obtained from X-ray projections; **(d)** Possible configurations based on subject position and X-ray source/collector orientations: 4 subject orientations, superior axis pointing toward the top of the device (I, II, III, IV) and 4 X-ray source/receiver directions (0°, 90°, 180°, –90°), yielding 16 possible configurations; **(e–g)** Example images for configurations of interest (II, 0°), (II, 90°), and (III, 90°).

Acquisition using the electronic portal imaging device for 2D radiographic images of the SARRP results in several possible configurations. An example of a phantom positioning configuration is shown in Figures 8a, b.

The phantom is positioned so that the axis pointing toward S aligns with the top of the device. From this position, the phantom can be rotated in the XZ plane, where the XYZ coordinate system for the device is defined in Figure 1. The X-ray source and detector are mounted on a rotating gantry, which can be rotated within the XY plane. This setup allows for multiple configurations. Using the net of the phantom cube, as shown in Figure 8c, the images obtained on the detector can be predicted as illustrated in Figure 8d. Configurations denoted by the tuple (C, θ), where *C*∈{I, II, III, IV} represents the phantom's position in the XZ plane, and θ represents the gantry rotation. Acquisitions from configurations (II, 90°), (III, 90°), and (II, 0°), highlighted in yellow in Figure 8d, produce images corresponding to coronal, sagittal, and axial projections, respectively, as shown in Figures 8e–g. Initially, a recording was performed with the phantom positioned posterior first in the device, resulting in the sequence (I, 0°), (I, 90°), (I, 180°), (I, –90°) (not shown). The use of the phantom facilitates the interpretation of the initial recorded images and enables the acquisition of the images presented here. These images correspond to the simplest configurations, requiring no additional processing for validation.

### 3.4 Acquisition using the 3.0 T whole-body MRI

**Figure 9:**
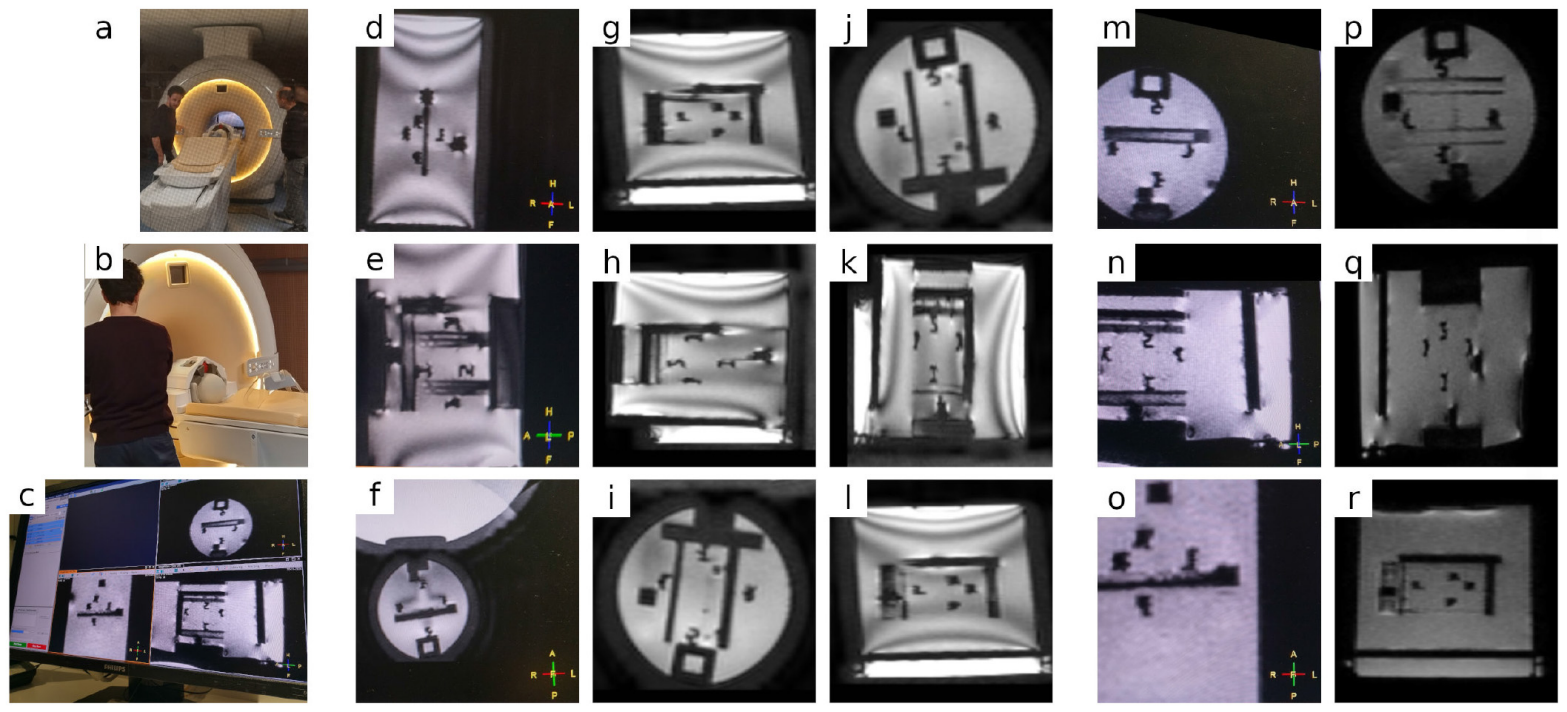
Acquisitions using a 3.0 T MRI for human imaging: **(a, b)** Phantom positioned in the device alongside a standard phantom to load coils and ensure proper pre-scan calibration; **(c)** Parameter settings and visualizations performed using the MRI scanner software; **(d–f)** Software views of the phantom positioned as a quadruped (head-first prone) using biped (head-first prone position) settings. Misalignment with the equipment coordinate system complicates evaluation. **(g–i)** DICOM to NIfTI export results in incorrect slice orientations; **(j–l)** Correct slice orientations achieved by modifying the affine transform of the NIfTI file; **(m–o)** MRI scanner views for the phantom in a head-first supine position. Axes align with the software coordinate system, though labels are symmetrically misplaced; **(p–r)** Unmodified export yields correct slice orientations.

This MRI scanner is primarily dedicated to human studies but may be used to study swines or primates. The phantom is first positioned in the prone position, mimicking a rodent introduced head-first into the device, as shown in Figures 9a, b.

The acquisition configuration is set for a human prone position. This results in the pre-visualizations shown in Figures 9d–f for coronal, longitudinal, and axial slices. The software uses a different convention, replacing the IS notation with FH (foot-head). The images are in agreement with an acquisition of a quadruped in a prone position (2) using parameters for a human prone (1). The coordinate substitution is as follows: PA(2) = IS(1) (FH(1)); LR(2) = LR(1), SI(2) = PA(1). Data are exported as a DICOM file and converted using dcm2niix to obtain an initial version of a NIfTI file. Visualization of the NIfTI file with MRIcron yields the images shown in Figures 9g–i. The permutation of IS and PA axes was required, as defined by the affine matrix *A*_2_ in Equation 1 to achieve the correct orientation, as illustrated in Figures 9j–l.

After repositioning the phantom in the device as a human supine, and performing an acquisition with the human supine configuration, the localization views are shown in Figures 9m–o. The coordinate system corresponds as follows: (LR(2) = LR(1), IS(2)=HF(1), PA(2)=PA(1)). However, the letters are displayed with axial inverted symmetry along the vertical axis of the visualized plane^6^. Exporting the data as a DICOM file and converting it with dcm2niix produces the MRIcron visualizations shown in Figures 9p–r. The slices are correctly oriented without requiring further transformations.

### 3.5 Summary of observations and automatic detection of flips and permutations

A summary of the visual and manual observations, corrections, and protocol adjustments for 4 out of 5 configurations is reported in Table 1.

**Table 1:** Summary of observations, corrections, and protocol adjustments across 5 configurations.

#	Mod.	Description	QC	Observations	Corrections
1	MRI T2	4.7 T for small animals	F	Wrong orientations detected using software programs *MRI file manager* and *nibabel.niftiImage.orthoview*.	Code patches applied to correct bugs.
2	X-ray CT	microCT for small animals	F	Wrong orientations detected using *dcm2niix* resulting in invalid NIfTI files.	Postprocessing after conversion using *nibabel* with affine transforms to obtain valid NIfTI files.
3	X-ray Image	Portal imaging device of the SARRP	F	Initial phantom positioning required postprocessing (rotations and axial symmetries).	Proposed improved configurations to obtain final images directly, without additional postprocessing.
4	MRI T1	3.0 T for human whole body acquisition	F	Phantom in quadruped head-first prone position – acquisition in human prone. Valid visualization in the device, but exported NIfTI files were invalid.	Postprocessing after conversion using *nibabel* with affine transforms to obtain valid NIfTI files.
5	MRI T1	3.0 T for human whole-body acquisition	.	Phantom in standard configuration: human head-first supine position – acquisition in standard mode. Valid visualization in the device and valid exported NIfTI files.	No correction.

#, Configuration index; Mod., Imaging modality; QC, Quality control status (F = failed, . = passed); Observations, Detected issues and error types; Corrections, Applied fixes or adjustments.

The automatic method is also evaluated on a set of 3D volumetric experiments. A summary of the results across different modalities is presented in Table 2. For each modality (Mod.), the parameters of the best configuration (Configuration), obtained with the cosine similarity score C, are compared with the affine transform of the initial volume at resolution S1 to determine the quality control (QC) reported in the table. Corrections are then applied based on this QC assessment. The time required to transform the initial volume to the template resolution (Time, first value) depends on the size of the low-resolution volume (S2). The second time value (Time, second value), required to detect flips, permutations, and translations, depends on the number of translations needed to align the low-resolution volume. The orientation correction process is fast and can likely be further optimized. With this computational method, every observation is reliably detected, reported, and corrected, even in acquisitions at the limits of spatial resolution (MRI T1, S1 resolution of (0.5, 1, 0.5)).

**Table 2:** Summary of the automatic detection of flips and permutations.

#	Mod.	S1	S2	Time	QC	Configuration	C
1a	MRI T2	((100, 100, 100), (0.5, 0.25, 0.5))	(41, 21, 41)	(24.0, 7.6)	F.....	((1, 0, 0), (1, 3, 2), (3, 7, 4))	0.76
1b	MRI T2	((128, 128, 128), (0.391, 0.391, 0.391))	(41, 41, 41)	(29.6, 0.5)	......	(1, 0, 0), (1, 3, 2), (4, 6, 5))	0.95
2	X-ray CT	((512, 512, 509), (0.076, 0.076, 0.076))	(32, 32, 31)	(15.3, 27.5)	F...FF	((0, 0, 0), (1, 3, 2), (7, 6, 5))	0.63
3	X-ray Image	n/a	n/a	n/a	n/a	n/a	
4	MRI T1	((512, 256, 512), (0.5, 1, 0.5))	(45, 53, 33)	(33.3, 6.93)	..F.FF	((0, 0, 1), (1, 3, 2), (7, 12, 0))	0.93
5	MRI T1	((512, 256, 512), (0.5, 1, 0.5))	(45, 45, 45)	(37.4, 24.9)	......	((0, 0, 0), (1, 2, 3), (9, 3, 8))	0.97

#, Configuration index; Mod., Imaging modality, 1a is for file formatted with MRI File Manager, 1b with brkraw; S1, size and resolution of High-resolution volume; S2, size of Low-resolution volume; Time, Subsampling and detection durations (in s); QC, Quality control status (F = failed, . = passed) reported for flips and permutations; Configuration, Best fit parameters (flips, permutations, translations); C, Cosine similarity score.

For example, the detection applied to configuration 1a corresponds to the parameters ((1, 0, 0), (1, 3, 2)). The matrices necessary to obtain the correct affine transform are, using notations of Section 2.4.3:


$$\begin{array}{l} B_{2}=\text{Π}F=\left(\begin{array}{ccc} 1 & 0 & 0\\ 0 & 0 & 1\\ 0 & 1 & 0 \end{array}\right)\left(\begin{array}{ccc} -1 & 0 & 0\\ 0 & 1 & 0\\ 0 & 0 & 1 \end{array}\right)=\left(\begin{array}{ccc} -1 & 0 & 0\\ 0 & 0 & 1\\ 0 & 1 & 0 \end{array}\right) \end{array}$$

$$\begin{array}{l} A_{1}=\left(\begin{array}{cccc} -0.5 & 0 & 0 & 21\\ 0 & -0.25 & 0 & 9.5\\ 0 & 0 & 0.5 & -24.75\\ 0 & 0 & 0 & 1 \end{array}\right)B_{1}=\left(\begin{array}{ccc} -1 & 0 & 0\\ 0 & -1 & 0\\ 0 & 0 & 1 \end{array}\right) \end{array}$$

$$\begin{array}{l} P=B_{2}B_{1}^{-1}=\left(\begin{array}{ccc} -1 & 0 & 0\\ 0 & 0 & 1\\ 0 & 1 & 0 \end{array}\right)\left(\begin{array}{ccc} -1 & 0 & 0\\ 0 & -1 & 0\\ 0 & 0 & 1 \end{array}\right)=\left(\begin{array}{ccc} 1 & 0 & 0\\ 0 & 0 & 1\\ 0 & -1 & 0 \end{array}\right) \end{array}$$

The corrected affine transform is then:


$$\begin{array}{r} A_{c}=A_{3}A_{1}=\left(\begin{array}{cccc} 1 & 0 & 0 & 0\\ 0 & 0 & 1 & 0\\ 0 & -1 & 0 & 0\\ 0 & 0 & 0 & 1 \end{array}\right)\left(\begin{array}{cccc} -0.5 & 0 & 0 & 21\\ 0 & -0.25 & 0 & 9.5\\ 0 & 0 & 0.5 & -24.75\\ 0 & 0 & 0 & 1 \end{array}\right)\\ =\left(\begin{array}{cccc} -0.5 & 0 & 0 & 21\\ 0 & 0 & 0.5 & -24.75\\ 0 & 0.25 & 0 & -9.5\\ 0 & 0 & 0 & 1 \end{array}\right) \end{array}$$

## 4 Discussion

We have developed a 3D-printed system that enables validation of orientations of 3D volumes and a computational method to correct affine transforms and align data with the RAS coordinate system. Together, these tools provide a dual approach to validating 3D volume orientations—through visual inspection at various stages of acquisition and processing, as well as computational optimization. This system can be tested once at the beginning of a dataset acquisition and used to validate the entire image processing pipeline. By experimenting with the phantom across different devices, we adjusted three conversion pipelines to export data into the NIfTI format with correct orientations and adjusted one protocol to obtain correct images without processing. This approach simplifies orientation verification and enhances comprehension of visualization at each step of image processing.

Glen et al. (2020) proposed a method to validate the lateralization of datasets by evaluating a goodness-of-fit score against known anatomical templates. While this method appears effective for published open datasets, its reliability is less evident for multicentric macaque datasets (Milham et al., 2018), where population control, particularly regarding individual lateralization, is limited. Additionally, it remains unclear whether the animals of these datasets are left-handed, right-handed, or ambidextrous and how templates are constructed for such a heterogeneous population. Recent studies suggest significant anatomical differences between left- and right-handed individuals (Hervé et al., 2006), further complicating the application of Glen et al.'s method to an uncontrolled population. In contrast, our phantom-based approach provides fiducial marks that allow for quick and unambiguous validation of the processing chain on a model, before applying pipelines to study subjects.

The acquisition using the 4.7 T MRI scanner for small animals validated the conversion to NIfTI format with brkraw, resolved one issue using our custom-developed software, and corrected a bug identified through nibabel (version 5.2.0) visualization. For this and other acquisition modalities, modifying the affine transform is one solution to ensure NIfTI complies with the RAS coordinate system convention. Alternatively, the data array defined by the ijk coordinate system can be transformed into a new i'j'k' order to match the RAS convention. However, this requires metadata updates to specify axis order during acquisition.

Acquisitions with the microCT X-ray scanner allowed us to adjust the pipeline to achieve validated orientations in NIfTI format. The phantom revealed that the equipment coordinate system did not match the one shown in Figure 1a. The CT scanner is designed to record small animals in a tail-first prone position along the LR, IS, and PA axes, resulting in the LSA convention consistent with DICOM standards. However, conversion with dcm2niix, version 1.0.20211006, was insufficient to obtain a correct NIfTI export for small animals acquisition, necessitating corrections using nibabel.

Acquisitions of 2D images with the SARRP portal imager enabled us to study different configurations and select the simplest ones to obtain the projections of interest. If the animal must be positioned differently (e.g., in decubitus), the phantom's cube net can help analyze configurations and projections.

Acquisitions using the 3.0 T MRI scanner for humans, despite the phantom's marker being at the resolution limit of the device, allowed us to propose corrections for studies involving medium-sized animals (e.g., Sus scrofa or Macaca) in non-optimal sphinx positions. While the phantom resolution is limited for human-scale devices, it remains effective for validating subject positioning and processing to produce standard-compliant NIfTI files. The phantom should be easily adaptable and scalable for compatibility with human imaging systems.

In both MRI scanners, the phantom's markers aligned with the software-displayed axes for standard positions (see Figures 6d–f, 9m–o), even though orientations followed the radiological convention: Coronal slices are given by looking in front of the anterior side of the subject. Sagittal slices are given by looking at the left side of the subject. Axial slices are given by looking in front of the inferior (feet) side of the subject. The prototype was designed to correspond to neurological convention views: Coronal slices, viewed from the posterior side. Sagittal slices, viewed from the right side; Axial slices, viewed from the superior (head) side. For radiological convention visualizations, the coordinate system remains consistent, but letters are axially symmetrized over the vertical axis. Correspondence between views can be established using the cube net (Figure 8c), with face pairs (1, 6), (2, 5), and (3, 7). Faces (1, 2, 3) follow the neurological convention, while (6, 5, 4) follow the radiological convention. A phantom adhering to the radiological convention can be easily created by: Replacing LR with RL in the first 3D-printed part; Replacing PA with AP in the second part; Replacing LR with RL in the third part; Adjusting the assembly accordingly. For bipedal subjects, the phantom can be scaled using computer-assisted design or 3D printing software and be oriented appropriately within the tube to trap air bubbles when filled with water, thereby minimizing MRI artifacts.

**Figure 10:**
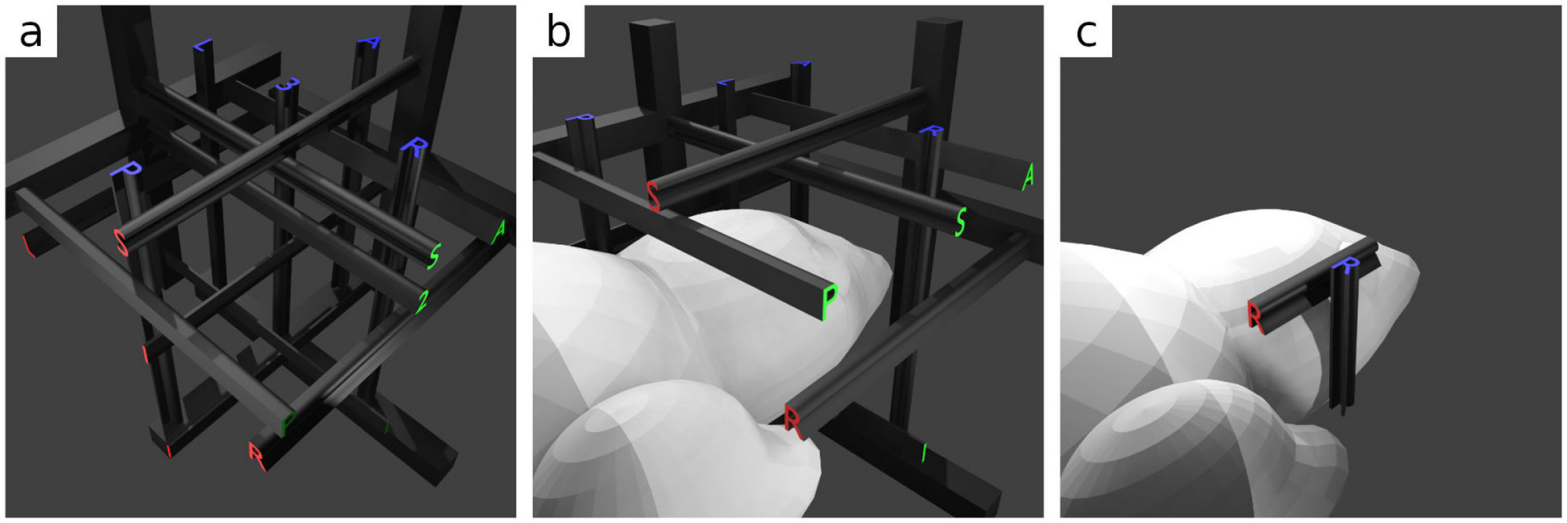
Others configurations for 3D printing: **(a)** Slice indices are printed on supplementary columns added in the center of the crossing support; **(b)** Empty spaces are reserved to position an object in the volume delimited by columns. **(c)** Only the problematic axes for LR are printed and embedded in the subject bed or placed on the animal.

We have chosen to place a symbol representing the slice index on the column that indicates the axis directions. This symbol, or any other slice identifier, can also be positioned alone at the center of each subsystem for every plane, as proposed in Figure 10a.

The system can also be modified to create a vacuum inside the columns, as illustrated in Figure 10b, in order to accommodate the head of a rodent, for example. With precise settings, markers, and calibration, this setup could be further refined to enable quantitative measurements or automatic registrations using the computational framework proposed in this study.

Additionally, the material properties of 3D-printed components, such as those studied for MRI in Sangal et al. (2023) and for CT scanning in Jusufbegović et al. (2022), can be explored to improve system susceptibility, reduce artifacts, or tailor the phantom for specific applications. Finally, the simple addition of LR columns to a mouse bed or other systems could be used or evaluated to control subject orientation, as proposed in Figure 10c. This reduced setup could be further tested for validation purposes and may offer better recognition compared to commercial fiducial markers, which typically consist of small dots. Additionally, it could be used for computational registration and positioning systems.

Through the diverse perspectives of the collaborators involved in this study, the phantom has not only established clear processes for data sharing, but it has also enhanced our understanding of the devices, practices, standards, and software we use, develop, or debug. Thus, it can be a valuable tool for practicing or testing new configurations and serves as an excellent tool for educational resources, helping students and new researchers grasp the complexities of the imaging pipeline.

## 5 Conclusion

In this study, we presented a 3D-printed phantom that can be easily fabricated by any staff working with MRI or CT scanners. This prototype allows for verification and validation of left-right orientations during both acquisition and data preprocessing. An automatic method to detect and correct flips and permutations has been proposed and evaluated. Given the complexity of the imaging parameters and the diversity of available 3D imaging devices, the phantom simplifies the development of pipelines to reorient data into the NIfTI format used in this study, which is critical for ensuring standardized dataset sharing within the research community. The framework is highly adaptable and can be tailored to other studies with different conventions, specific orientations, subject positions, or embedding requirements.

## Data Availability

The datasets, the 3D models, the STL Files for 3D printing, and the codes for the automatic detection of flips and permutations, as well as correcting them, presented in this study can be found in online repositories. The names of the repository/repositories and accession number(s) can be found below: https://doi.org/10.18112/openneuro.ds006123.v1.0.0; https://doi.org/10.5281/zenodo.15211130; https://github.com/gjpcbecq/phantom3D.git.

## Footnotes

1 Dimensions are given in meters into Blender. The STL export converts 1 unit to 1 mm, with the transformation *scale* parameter set to 1 and the *Scene Unit* option unchecked.

2 They correspond to the Chinese/Japanese numerals 1 , 2 , 3 , but are easier to 3D print.

3 A volume of 30 mm × 30 mm × 30 mm at an isotropic cubic resolution of 0.1 mm contains 300^3^ = 2.7*e*7 voxels.

4 A volume of 30 mm × 30 mm × 30 mm at an isotropic cubic resolution of 1.25 mm contains 24^3^ = 13, 824 ≈ 1*e*4 voxels.

5 DICOM, or veterinary conventions(Veterinaria, 2017).

6 This discrepancy arises because the radiological convention presents an image as if viewed from the front of the plane of interest, while the neurological convention presents an image as if viewed from behind the plane of interest. This inversion can be resolved using the cube net shown in Figure 8c.
